# Breast Imaging: How We Manage Diagnostic Technology at a Multidisciplinary Breast Center

**DOI:** 10.1155/2012/213421

**Published:** 2012-07-05

**Authors:** Alejandro Tejerina Bernal, Antonio Tejerina Bernal, Francisco Rabadán Doreste, Ana De Lara González, Juan Antonio Roselló Llerena, Armando Tejerina Gómez

**Affiliations:** Centro de Patología de la Mama, Fundación Tejerina, José Abascal 40, 28003 Madrid, Spain

## Abstract

This paper discusses the most important aspects and problems related to the management of breast cancer imaging, at a center specialized in breast pathology. We review the established and emerging diagnostic techniques, their indications, and peculiarities: digital mammography, CAD systems, and the recent digital breast tomosynthesis, ultrasound and complementary elastography, molecular imaging techniques, magnetic resonance imaging, advanced sequences (diffusion), and positron emission mammography (PEM). The adequate integration and rational management of these techniques is essential, but this is not always easy, in order to achieve a successful diagnosis.

## 1. Introduction

Breast cancer remains the most prevalent cancer disease in women of developed countries with great social and economic impact. For all these reasons, the scientific community concentrates on improving imaging methods, developing drugs for new therapeutic targets, and working at well-coordinated multidisciplinary units.

The Breast Pathology Center of Madrid has more than 40 years of experience in this field, with over 60 medical professionals working with and for breast pathology. More than 50,000 patients are examined each year, and over 400 new cancers are diagnosed annually.

Historically, the first treatment which changed the course of the disease was Halsted's radical mastectomy [1], in the late nineteenth century. Many subsequent advances took place, but what really marked a turning point was the idea that a systematic study with mammography, at highest risk ages, could detect and, therefore, change the evolution of breast cancer in population groups.

Imaging techniques have experienced a highly significant development in recent years. The morphological image, of great value, has evolved into a physiological and functional image, capable of providing additional information, very valuable to better understand disease processes. Many technological changes have been established since the development of the first analogue mammography device in the sixties, with which we started working but, undoubtedly, the most important advance has been the introduction of digital mammography.

Ultrasound use increased in relevance over the years as well as its application in the remaining organs and systems, while the image improved with the first static ultrasound devices, nowadays becoming a daily practice routine.

Magnetic resonance imaging (MRI) is at present an almost indispensable technique for local staging and followup of breast cancer, although many technical improvements have been necessary for this imaging test to provide adequate sensitivity and specificity to become widely used.

Thanks to the merging between morphological and molecular techniques, PET-CT has gained importance in cancer staging, including breast cancer, always being open to other specific nuclear medicine studies dealing with the study of the breast, as in the PEM technique, which is beginning to evolve, even though the first clinical studies show very satisfactory results.

This whole technological arsenal must be integrated into breast diagnostic imaging units which, in turn, belong to multidisciplinary breast pathology units. The professionals must be responsible for knowing its extent, indications and limitations, in order to achieve the highest diagnostic performance and, ultimately, an early diagnosis.

## 2. Digital Mammography and Its Resulting Techniques

### 2.1. Digital Mammography

Mammography must be an effective universal technique, reproducible, with enough sensitivity and specificity to ensure early detection and influence the course of the disease, decreasing mortality by 20–30% [2].

So, it is the “gold standard” technique for the study of breast pathology and is used in screening studies in women aged between 40–50 years, depending on each country. 

In 2000, the Food and Drug Administration (FDA) approved the usage of digital mammography (DM) for diagnostic use, with consequent improvements in image resolution and digital manipulation and storage. These aspects make it a more widespread technology, being practically an essential prerequisite for a breast diagnostic center. In Spain, we became leaders of this technique, which involved time to adapt and change, as the file storage systems (PACS-RIS) as well as the workstations had to be modified.

The DM can be classified into two large groups depending on its usage. Detectors called computed radiography (CR) belong to the first group. These have the advantage of being employed with conventional mammography devices. The second group of detectors (DR) encompasses all those which are integrated within a digital mammography system itself.

At present, there is no scientific doubt with respect to the usefulness of mammography or diagnostic mammography for breast cancer early detection programs, and an improvement in its effectiveness is expected when using new techniques (CAD, tomosynthesis, ultrasound, etc.), which increase the sensitivity and specificity of this imaging test.

### 2.2. Digital Breast Tomosynthesis (DBT)

Mammography continues to have certain limitations inherent to the principle of obtaining a two-dimensional (2D) image of a three-dimensional compressed glandular parenchyma, causing lesions to be masked sometimes due to the superimposition of glandular structures in the X-ray beam.

**Figure 1:**
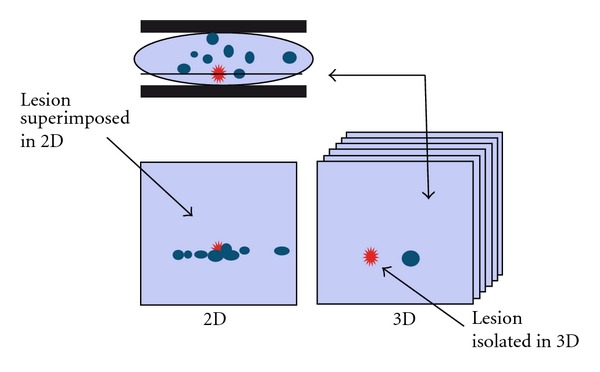
This figure shows, in a 2D mammography, a potentially malignant lesion, in red. This lesion can remain occult between benign lesions, such as cysts represented in blue. However, at the selected tomographic slice, the malignant lesion is visible avoiding the superimposition.

This structure superimposition can impede visualizing a lesion (false negative) or identifying a lesion as suspicious which finally is a glandular accumulation (false positive) (Figure 1).

Based on this fact, Digital Breast Tomosynthesis arises (DBT). In 2010, we incorporated this new technology at our center, being the first Spanish center and one of the first European centers to work with DBT.

**Figure 2:**
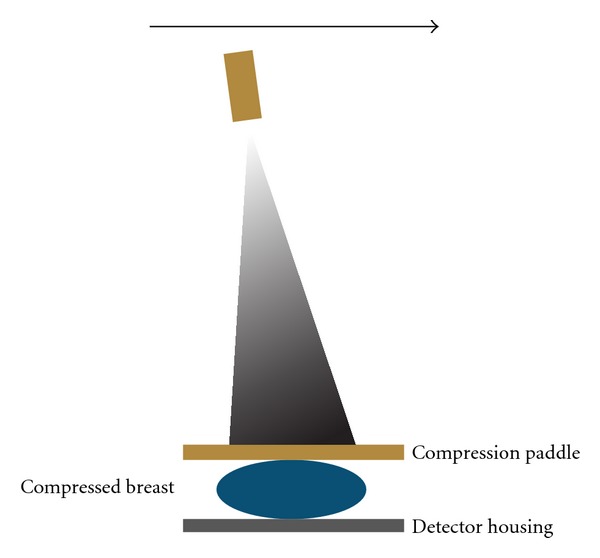
Tomographic projection obtention system using rotatory X-ray tube.

It is a mammography device which uses 3D technology and a rotary head tube, performing different projections of a static breast (Figure 2) with a specified angle (between 15–45°) and employing two types of technology: “continuous” or “step and shoot,” depending on the commercial device which is available.

The projections can be obtained craniocaudally (CC) and oblique-medial-laterally (OML), except for some commercial devices which only conduct OML projections.

Then, the images are reconstructed at a cutting thickness between 0.5–1 mm, and they can be visualized at a workstation, with software specialized in tomosynthesis, individually or as a “film,” as which is displayed with a conventional scanner. So that a compressed breast, 5 cm thick, will generate 50 tomosynthesis images 1 mm thick.

In this way this is a technique which, conceptually, would be a combination between mammography and scanner and it can be considered a tomographic application of digital mammography.

The radiation dose of a 3D tomosynthesis and 2D mammography study is within the standards accepted by the Mammography Quality Standards Act (MQSA), although the radiation dose is somewhat larger than that generated by the current modern mammography devices with tungsten tubes.

The acquisition time of the study must be low in order to reduce the possibility of motion artifacts and vascular stasis images, due to the elevated compression time, as well as to avoid a prolonged study time which causes discomfort to the patients.

In our case, we always use DBT as a complement to the DM and never as a single study without mammography. After an initial adaptation and training phase in reading these new dynamic images, we conclude that DBT provides an enormous potential as it avoids image overlapping with respect to DM.

**Figure 3:**
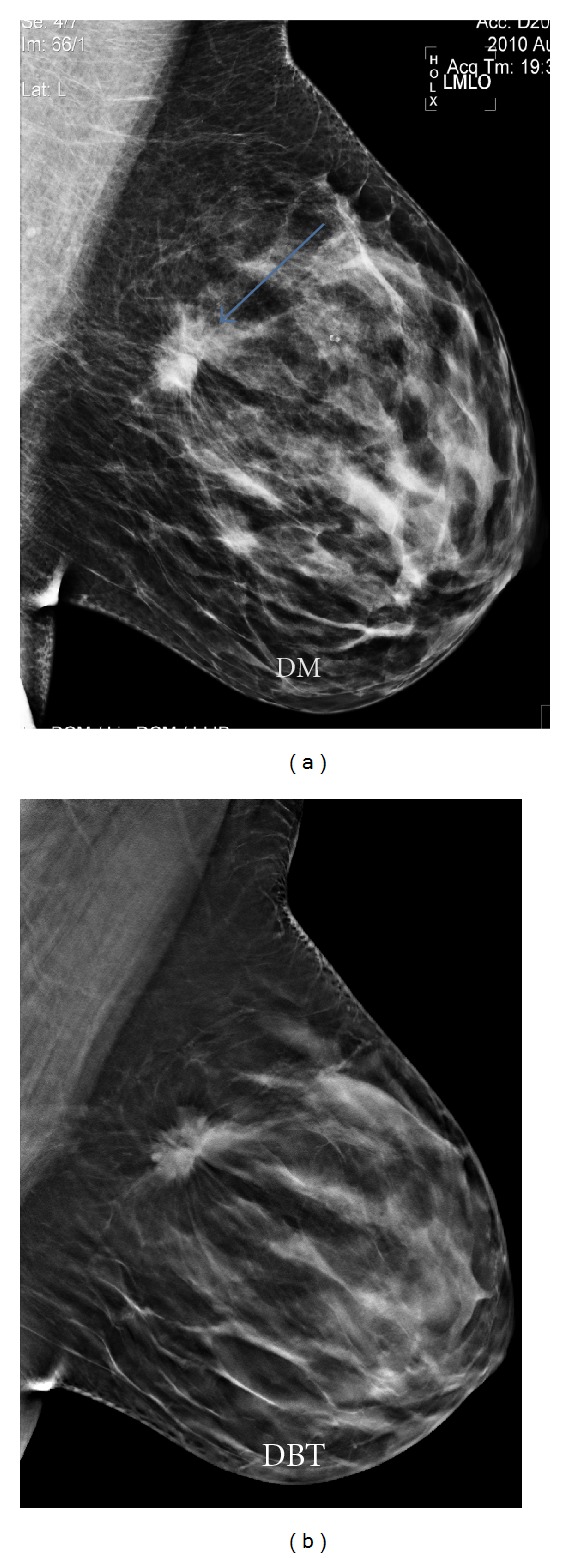
Lump detected more clearly with the DBT than the DM technique, visualizing better its margins, helping to catalogue it as an image highly suggestive of malignancy, BI-RADS 5.

For us, the main advantages of carrying out a DBT study, complementary to DM, are therefore based on the following principles:

- it improves the display of the lump contour [3], helping their characterization and making a more accurate estimation of the BI-RADS classification (Figure 3);
- it helps detecting distortions, so far hidden in certain mammographies;
- it reduces the number of false positives corresponding to glandular clusters;
- it avoids carrying out a large number of special projections, except for cases where the only findings are microcalcifications [4];
- it provides greater security in the study of dense breasts;
- it reduces the number of recalls at screening mammography [5];
- with respect to microcalcification determination, recent studies suggest that it is similar to mammography, even though it provides valuable information as to location and specific provision.

Although its usefulness is undeniable, it is still early, and there are some unresolved issues. There are no accepted protocols for use with respect to whether it must be used in all patients or not, or if both projections must be carried out in each breast always or if it is possible to use tomosynthesis without mammography.

**Figure 4:**
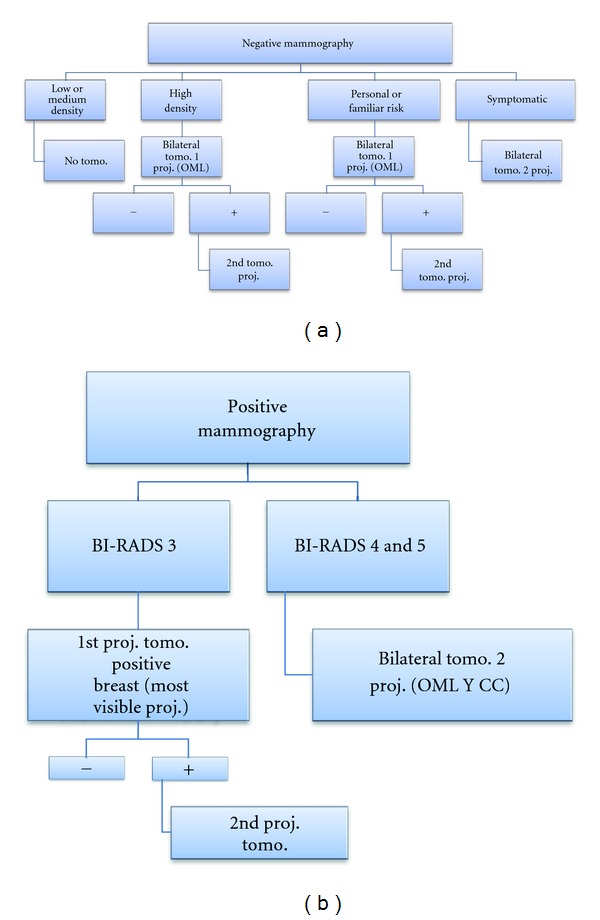
Tomosynthesis usage protocol in case of negative or positive mammography.

Our group presented protocols for optimal use [6], based on mammographic findings, breast density, clinical symptoms, and background information, which are summarized in the diagrams shown in Figure 4. 

For all these reasons, this technique is spreading across European and North American centers, including it in routine diagnostic practice, and there are ongoing studies designed to analyze its role in breast cancer screening.

### 2.3. Computer-Aided Detection (CAD) Technology for Mammography Reading

The sensitivity and specificity of mammography vary between 70–96% and between 90–95%, respectively [7, 8]. The reasons for this variability lie in the quality of this technique and the individual ability of the radiologist. Several clinical studies have demonstrated that between 30–70% of cancers, diagnosed during a screening program, can be seen on mammograms previously read as normal; in half of the cases, this is due to detection errors and, in the other half, to reading errors [9]. In order to try to overcome these disadvantages, there are different reading techniques. That with best performance appears to be the “double reading,” and it is the one used routinely in our center. In recent years, several options have emerged, such as the integration of computer-aided detection systems (CAD) for mammography reading.

These computer systems have been designed as tools to support the radiologist in the detection of suspicious lesions for breast cancer. The most developed systems are CAD systems at present, and they are mainly used for screening.

The sensitivity of these systems varies with the type of lesion, and there seems to be higher sensitivity levels in microcalcifications than in detecting lumps or other findings [10].

Some of the disadvantages of CAD systems are their low specificity, the radiologist having to reject false positives detected by the system, creating a lack of confidence, especially in nonexpert radiologists. Furthermore, this may lead to increased reading times, this fact being important when reading screening mammographies.

However, CAD systems, as well as other technologies, are improving significantly, and new CAD systems have emerged even to evaluate tomosynthesis.

For this reason and in our experience, the main advantage of the use of computer-aided systems is its application in screening mammography, mainly for detecting microcalcifications, provided that they are used by mammography expert radiologists.

### 2.4. Location Systems and Radiological Stereotactic Biopsy

There are at present two groups of stereotactic localization systems which are used for preoperative localization by wire or for biopsy, by means of the core biopsy or vacuum-assisted biopsy (VAB) system, the latter being used more often radiologically guided.

They consist on systems which use a prone table and systems with a vertical or lateral stereotaxic device, aided by a mammography unit, which will be used interchangeably, based on availability of the technique in each center. Except for certain cases, if both systems are available, it seems preferable to use the prone table for biopsies as it is more comfortable for the patient. For preoperative localization, it will depend more on the availability, at the center, of a mammography device or a prone table, if it were being used at that time for biopsy or for mammography workout.

Additionally, nowadays the assisted biopsy system for tomosynthesis has emerged as several images, though only few, are visible only with this technique, needing guided biopsy by this system. Nevertheless, until we disposed of this type of assisted biopsy system for tomosynthesis, we resolved this problem using a fenestrated compression device, and we were guided by the depth in millimeter slices provided by tomosynthesis, to calculate the exact spot and, in such a way, to be able to place a preoperative wire or to carry out core biopsy although, in order to carry out VAB, we did not dispose of any support, for which it was impossible to conduct.

The type of subsidiary images of radiological biopsies will preferably be microcalcifications, distortions, and focal densities with little if any translation with other imaging techniques.

Sometimes it is necessary to place a metallic marker postbiopsy to serve as a guide for further surgery if necessary.

## 3. Ultrasound: Elastosonography

Ultrasound is the essential complementary technique for mammography and, in some cases, as in young or pregnant patients, it is the first choice.

Ultrasound systems have improved greatly with the introduction of high-frequency probes, between 12–15 MHz, harmonic images, and 3D images which provide a very high-quality morphological image of the surface. Ultrasound, when used as the first diagnostic technique, has two major disadvantages: the microcalcifications are difficult to detect by ultrasound; it is a technique which depends on the browser, and this can take a very long time. For these reasons, this technique must not be used initially, although it has many indications and utilities.

Elastosonography is a step forward as functional technique.

In a few years time, it has moved from being a technique mainly used to differentiate between cystic and solid images, to be an indispensable complement, establishing the degree of suspicion of a breast lesion not determined by mammography.

As advantages over mammography, we can include its ability to assess the internal structure of lesions in multiple planes, their orientation, morphology and margins, both in predominantly fatty breasts and, above all, in breasts with dense glandular structure, where the mammography is more limited.

### 3.1. Ultrasound Indications

Breast ultrasound, as initial workup for breast pathology, can be indicated in some cases.

In young women, below 30 years old, with dense breasts and symptomatic, ultrasound constitutes the technique of choice as well as in pregnant or breastfeeding women. In cases of inflammatory disease, ultrasound is better tolerated and superior to mammography when identifying collections. In women with mastectomies, it is used to study the surgical site and to search for recurrences or possible surgery complications.

Ultrasound, as a complementary technique to mammography, has a key role in many cases.

To analyze mammographic findings, establishing a BI-RADS ultrasound staging [11]. To examine breast implants although, if there is any doubt, a MR should be carried out. To visualize galactophoric trees in patients with secretions. For negative mammogram and palpable lesion cases, ultrasound plays a crucial role as well as in women with risk factors and dense breasts [12]. For malignancy confirmed cases, ultrasound provides important information about tumor size, pectoral and skin involvement, multifocality and multicentricity, and axillary staging, although its performance is higher when combined with MRI.

**Figure 5:**
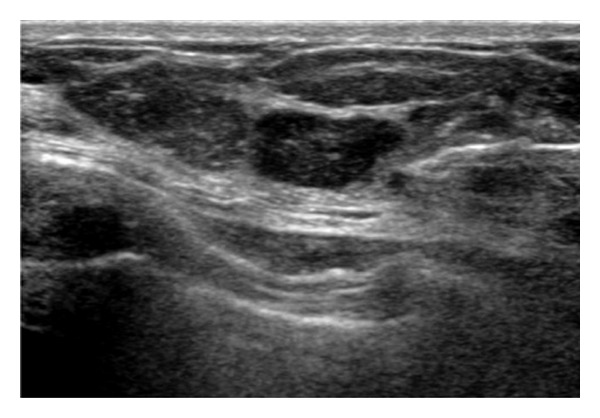
Ultrasound-guided biopsy of a lump from a patient with implant, allowing monitoring at all times the progress of the tip of the needle.

Ultrasound, as a guide for interventional procedures and preoperative marking, constitutes another mainstay, being the method to be used due to its comfort, lower cost than with other techniques, and increased safety for the patient, as it monitors at all times the progress of the tip of the needle (Figure 5).

For this reason, whenever a lesion is visible by ultrasound, except for certain cases, it is the method we use for both FNAB or core biopsy. You can also use vacuum-assisted biopsies (VABs), although it is less comfortable due to the greater weight of these devices, its use being limited practically to cases of excision of benign lesions, mainly fibroadenomas or papillomas. And, although it is an accepted technique, it continues to raise controversy at present and, in our experience, its application is limited to very specific cases.

### 3.2. Elastosonography: A Functional Technique

It is a recent ultrasonic technique based on the same principle as breast palpation, that is, the estimation of the consistency or hardness of the tissues.

It is a comfortable technique, as it does not require more than the same transducer which has been employed for B-mode images, and it is quick as it does not extend the test more than a couple of minutes.

At present, there are two types of sonoelastography: compression elastography and supersonic elastography.

**Figure 6:**
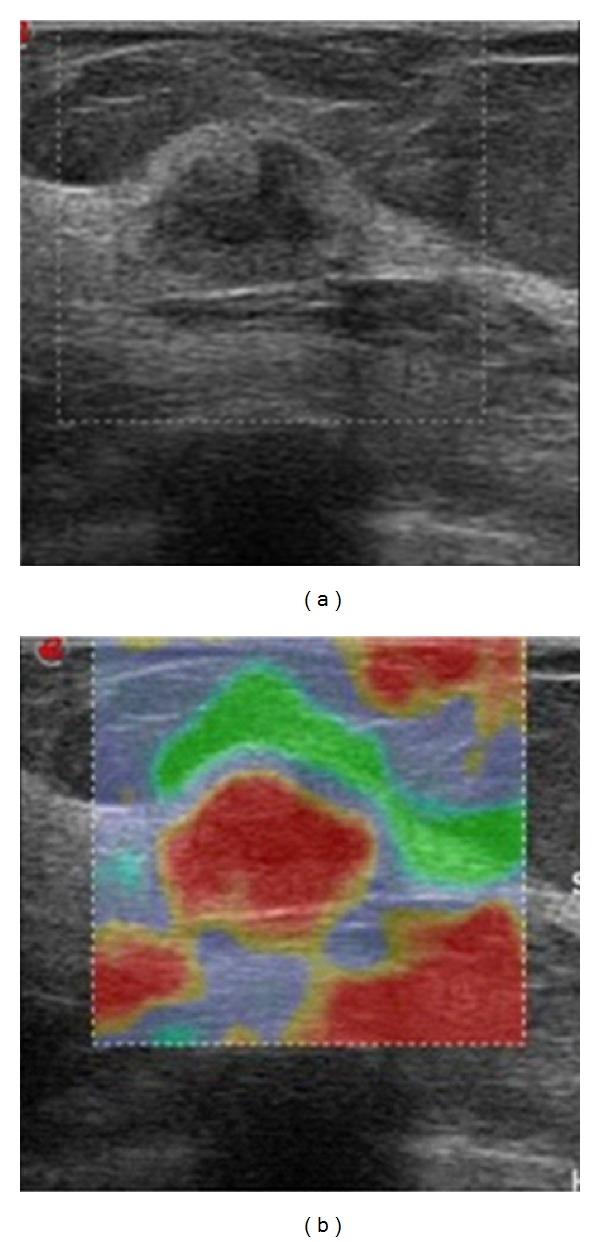
Comparative B-mode ultrasound image of an unspecified lesion which, on the elastographic map, corresponds to an increased hardness lesion, shown in red. It corresponded to invasive ductal carcinoma.

Compression elastography (*strain imaging*) assesses the deformation of different tissues as a result of the effect of compression waves emitted from the transducer through which we gently compress the skin. This technique is qualitative or semiquantitative, and its results are reflected in two types of elastograms. One of them is based on a gray scale, where the softer tissues appear in white, while the hardest appear in black. Once we differentiate the lesion to examine, which will appear darker than the adjacent tissue structures, we must take a look at the lesion size. Benign lesions tend to have a resulting image smaller than the B-mode image. By contrast, malignant lesions have a greater or equal resulting image than the B-mode image. This phenomenon may be related, according to Insana et al. [13], to the desmoplastic reaction present in most of these lesions. The other elastogram is characterized by presenting in colors both the lesion and the surrounding tissue, depending on their elasticity and according to a color scale that varies depending on the commercial firm (Figure 6). Itoh's group [14] described an elasticity chromatic scale which classified elastographic findings in a manner similar to that used by the BI-RADS classification in B-mode.

Supersonic elastography (*supersonic shear waves imaging*), developed more recently than the previous one, is characterized by the use of transmitted ultrasound pulses at high speed from the same transducer and without compressing the skin. The tissues generate waves in response to these pulses that allow you to know different parameters quantitatively, such as maximum, minimum, or average elasticity of the different structures studied.

Whatever the elostographic system we have available, this technique must be understood as a value added to the B-mode ultrasound. A value that, although being rare in the case of lesions with an ultrasound BI-RADS 2, 4b, 4c, or 5, that is, in those lesions with high probability of benignancy or malignancy, it can be considerable in the case of more uncertain lesions, such as BI-RADS 3 (probably benign) or BI-RADS 4a (low malignancy suspicion). So that this technique, used as complement to B-mode ultrasound in the study of BI-RADS 3 lesions, will be capable of confirming the ultrasound hypothesis of benignancy, thereby reducing the number of unnecessary biopsies and guiding these low-risk patients to a followup with greater safety [15].

## 4. Magnetic Resonance Imaging: New Advances

Magnetic resonance imaging is a less available than the previously described methodologies, but this does not mean that it has to remain in the background, as it is of great diagnostic value in many cases.

Since the Introduction of contrast agents, improved antenna surface and the development of new imaging protocols, MRI emerged as a promising technique for the detection, diagnosis, and staging of breast cancer.

Its introduction as an imaging test in the field of breast cancer has implied a huge progress due to its high diagnostic performance and ability to detect tumor burden and other pathological conditions, both in morphological and functional terms.

MRI has a number of morphological sequences, highlighting T2 FSE, STIR, and T1, which allow us to evaluate breast tissue density and morphological changes and to assess the condition of the skin, armpits, and the edge of the pectoral muscle.

Having obtained the morphological sequences, the contrast agent is administered in order to assess, by subtraction sequences, MIP and MPR, the uptake of the breast, the possible tumor uptake, and we will evaluate the dynamic sequences, by means of the time/intensity curves.

The perfusion, spectroscopy, and diffusion sequences have not been validated at a large scale and, consequently, they must be conceived as a complement to the sequences described above, while providing a promising future.

Given the high false positive rates produced by this technique, in patients staged by MRI, and based on the fact that additional tumor burden diagnosed by MRI can be treated effectively with adjuvant chemotherapy and radiotherapy, as was demonstrated by Fisher et al.'s and Veronesi et al.'s clinical trials [16, 17], all this leads to state that this technique has its detractors maintaining that any additional lesion diagnosed by MRI does not have real impact on patient's survival.

A meta-analysis by Houssami and Hayes [18] analyzed the rate of additional disease diagnosed by MRI (16%), and the proportion of patients in which surgical planning was altered (7.8–33%).

The heterogeneity of the literature is due to the fact that, in real clinical practice, not all radiologists work in a multimodality environment, handling all the techniques in harmony and integrating their clinical and radiological breast cancer knowledge. This is reflected by the rate of additional lesions detected by MRI before surgery, making the surgeon and oncologist to distrust this technique.

### 4.1. Indications for MRI

There are at present some clear indications accepted by the scientific community: to evaluate response in patients treated with chemotherapy, screen high-risk patients, detect primary tumors in patients with nodal metastases of unknown character, and analyze breast implants to rule out rupture. However, there are some clinical applications that are not accepted by all the professionals, the main one being breast cancer staging but, nonetheless, it is already included in routine clinical practice at multiple medical centers and hospitals throughout the world.

**Figure 7:**
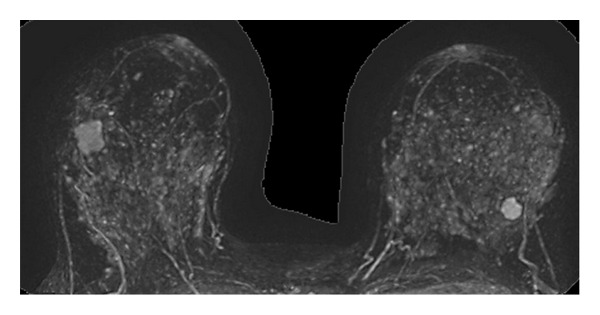
MIP RMI reconstruction. Preoperative staging study due to a right breast lesion, where a second lesion was identified at the left breast, corresponding to synchronic bilateral carcinoma.

Breast cancer staging is essential to establish the type of treatment for the patient and to assess tumor size, the presence of multifocal or bilateral lesions and the expansion to the nipple-areola complex or intraductal component (Figure 7).

In the case of detecting an additional lesion, it will always be necessary to perform a “second-look” ultrasound in the area and to carry out a biopsy of this lesion if it were visible. Our experience is that around 80% of lesions, detected by MRI and not previously identified by conventional methods, are detected by a second-look ultrasound, thereby limiting MRI guided biopsies to very specific cases, ductal enhancements for the most part. Some of the criteria to be analyzed, in order to establish successful staging, will be the percentage change of therapeutic attitude, reexcision rates, and recurrence rates.

**Figure 8:**
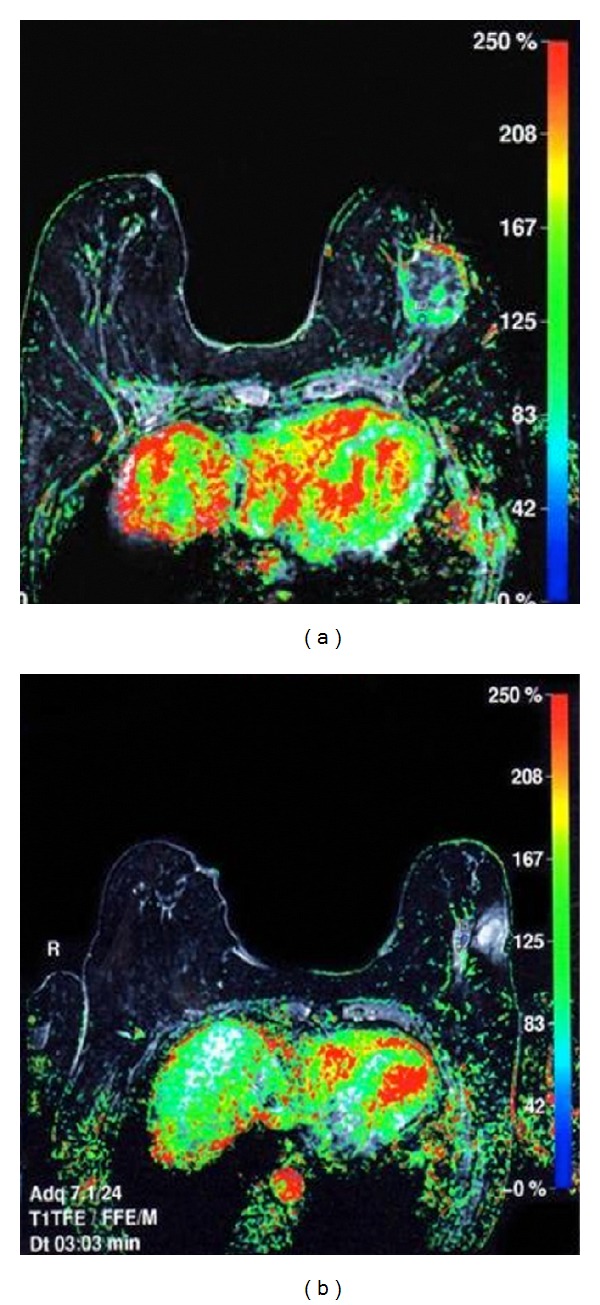
MRI during neoadjuvant treatment monitoring, where one can visualize macroscopic tumor disappearance corresponding to greater partial response.

Evaluating response to treatment produced by primary chemotherapy is of great importance since it is an *in vivo* chemosensitivity test, allowing varying cancer treatment if not effective. MRI enables establishing the response rate quantifying tumor shrinkage volume (Figure 8) and permitting the oncologist to change the type of neoadjuvant drug in the middle of treatment or to decide at the end of it if it possible to conduct conservative surgical treatment. Nevertheless, there is some tumor under- and over-estimation, a fact largely being solved thanks to diffusion sequences and spectroscopy.

The diagnosis of tumor recurrence is uncommon during the first 18 months after treatment and, in most cases it appears at the surgical bed for the first 5 years. We prefer carrying out MRI after the first 12–18 months of treatment, in order to avoid the false positives caused by fat necrosis and inflammatory component. Even though, if in our practice we detect a high suspicion of recurrence, we conduct MRI whatever the time elapsed since surgery.

**Screening High-Risk Patients**

Cancers of patients with BRCA 1 and 2 mutations have particular characteristics. These tumors have well-defined margins, apparently benign, and appearing in very young patients, with a much shorter doubling time than in noncarrier patients, and the most prevalent molecular phenotype has the worst outcome, triple negative. The mammography has less value in these patients as they often have dense breasts due to their youth. It is estimated that the heterofamily factors are among the factors responsible for approximately 10–15% of cases. Several multicenter studies [19] show that the sensitivity of following these patients with MRI is more than twice that with mammography only. For this reason, we prefer following these patients with annual MRI.

**Search for Occult Breast Cancer**

Occult breast cancer represents less than 1% of all breast cancers. MRI has emerged as the technique of choice before taking any therapeutic approach.

**Evaluating Patients with Breast Implants**

**Figure 9:**
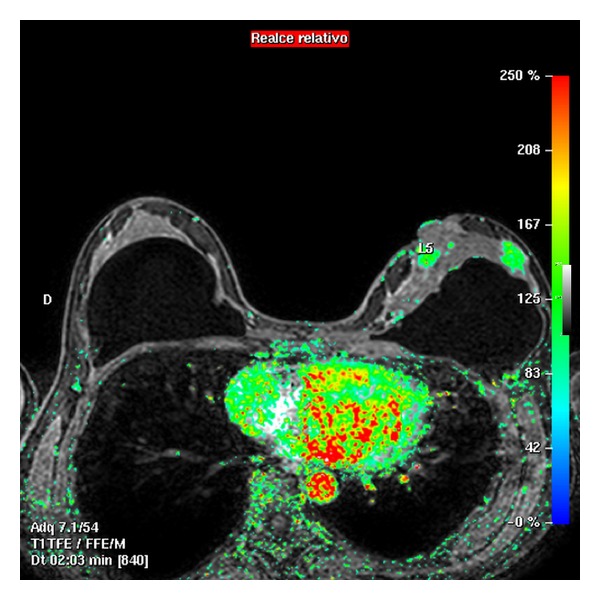
Tumor recurrence at a previously mastectomized patient with bicameral implant.

In this case, MRI plays two key roles: implant rupture detection and cancer detection in patients with implants, which hampers visualizing breast tissue with other types of techniques (Figure 9). It is true that ultrasound has a good sensitivity and specificity also for evaluating implants and can be used as the first technique, leaving MRI for cases of diagnostic uncertainty and for implants and family genetic burden cases.

### 4.2. Advanced Sequences: Diffusion

Diffusion sequences are based on the principle of mobility of water molecules in a medium. Thus, a high-cellularity structure will have a lower diffusion of its water component due to the high number of cellular elements.

So the diffusion-MRI can be used to calculate the apparent diffusion coefficient (ADC), which is a quantitative measure of water diffusion, providing information about tumor cellularity and membrane integrity, and it is sensitive to the intratumoral changes produced by chemotherapy treatment.

The calculation of ADC can be used as an additional element in the evaluation of a breast lump, joining the established criteria, such as margins, shape, signal characteristics, tumor enhancement, and dynamic study. Apart from its main use, which has been studied many times, and the use of this parameter as a predictor of neoadjuvant treatment response, it can assist in individualized treatment plans and avoid ineffective chemotherapy.

## 5. PET-CT and PEM

With the merging between morphological and functional techniques, PET-CT studies have become a benchmark for the diagnosis and followup of cancer diseases.

They are used for head and neck tumors, thyroid follicular cancer, single pulmonary nodules, lung cancer, esophageal cancer, colorectal cancer, lymphoma, melanoma, and breast cancer of course.

The metabolic activity of neoplastic tissue, provided by ET, offers additional information about tumor biology and can be used to differentiate between benign and malignant, metastasis identification in early stage for disease staging, treatment response, and tumor aggressiveness.

In breast cancer study, whole-body PET-CT is extremely useful in tumor staging at a distance, especially in advanced stage breast cancer cases and (plays) has a role in locoregional lymph node staging.

However, for breast cancer imaging, it has an important spatial limitation, their sensitivity and specificity figures being 80 and 76%, respectively [20].

### 5.1. Positron Emission Mammography (PEM)

Due to the limited resolution of PET equipment and the space limitations of the current protocols for CT acquisition, small-size breast tumors are not visible using this technique, until they reach a certain size, and they can be visualized with other techniques like MRI. This is what has led to the development of a PET device dedicated to the breast, PEM (positron emission mammography).

The high-resolution PET has been designed to detect small hypermetabolic lesions of external parts, such as the breast.

There are several commercial firms which have developed the PEM and, although they are still in early clinical development, initial results show sensitivity levels of 93% [21], similar to MRI and also high specificity levels 93% [22] higher than MRI.

These results position this technique as promising, with applications in surgical planning, monitoring response to neoadjuvant treatment and recurrence. However, there are some disadvantages related to the cost and technical difficulty of handling radiopharmaceuticals. Another issue to resolve is which will be the interrelation between MRI and PET and if a coexistence of both techniques is possible or if one of them will prevail over the other. It is still too early, and only time will give us the answer.

## 6. Conclusion

The constant technological innovation is undeniable both in improving the morphological and the functional techniques. There is a very narrow common future, in which the combined techniques are becoming a present reality for the diagnostic imaging of breast pathology, led by professionals with an increasing degree of specialization.

Nowadays, there are many technological innovations available to us, but it would not be beneficial that, instigated by the novelty, we lost the true meaning of each technique, and if we anticipated issues with no scientific evidence still.

Nonetheless, we must have an open mind to integrate the new emerging techniques with the established ones in order to take advantage of the combination of both.

It is, therefore, important that image specialists, dedicated to breast pathology, work in a multimodal environment, managing the main techniques and keeping abreast always.

Working in multidisciplinary units composed of clinicians, surgeons, oncologists, imaging specialists, and so forth, is essential to allow progress in breast cancer treatment and diagnosis.
